# Clinical Investigation on the Impact of Cannabis Abuse on Thyroid Hormones and Associated Psychiatric Manifestations in the Male Population

**DOI:** 10.3389/fpsyt.2021.730388

**Published:** 2021-12-03

**Authors:** Anum Muzaffar, Sami Ullah, Fazal Subhan, Zahid Nazar, Syed Mehdi Hussain, Fazli Khuda, Abuzar Khan, Ameer Khusro, Muhammad Umar Khayam Sahibzada, Sarah Albogami, Ahmed M. El-Shehawi, Talha Bin Emran, Binish Javed, Javed Ali

**Affiliations:** ^1^Department of Pharmacy, University of Peshawar, Peshawar, Pakistan; ^2^Department of Psychiatry, Lady Reading Hospital MTI Peshawar, Peshawar, Pakistan; ^3^Syed's Clinic for Psychiatric Illness, Peshawar, Pakistan; ^4^Research Department of Plant Biology and Biotechnology, Loyola College, Chennai, India; ^5^Department of Pharmacy, Sarhad University of Science and Information Technology, Peshawar, Pakistan; ^6^Department of Biotechnology, College of Science, Taif University, Taif, Saudi Arabia; ^7^Department of Pharmacy, BGC Trust University Bangladesh, Chittagong, Bangladesh; ^8^Atal Bihari Vajpayee Institute of Medical Sciences, Dr. Ram Manohar Lohia Hospital, New Delhi, India; ^9^Department of Pharmaceutics, School of Pharmaceutical Education and Research, Jamia Hamdard, New Delhi, India

**Keywords:** cannabis, thyroid hormones, psychiatric symptoms, cardiovascular parameters, dependence

## Abstract

Cannabis abuse is a common public health issue and may lead to considerable adverse effects. Along with other effects, the dependence on cannabis consumption is a serious problem which has significant consequences on biochemical and clinical symptoms. This study intends to evaluate the harmful effects of the use of cannabis on thyroid hormonal levels, cardiovascular indicators, and psychotic symptoms in the included patients. This prospective multicenter study was conducted on cannabis-dependent patients with psychotic symptoms (*n* = 40) vs. healthy control subjects (*n* = 40). All participants were evaluated for psychiatric, biochemical, and cardiovascular physiological effects. Patients were selected through Diagnostic and Statistical Manual of Mental Disorders (DSM)-IV criteria and urine samples, exclusively for the evaluation of cannabis presence. Serum thyroid stimulating hormone (TSH), T3, and T4 levels were measured using the immunoassay technique. Patients were assessed for severity of depressive, schizophrenic, and manic symptoms using international ranking scales. Various quantifiable factors were also measured for the development of tolerance by cannabis. Among the patients of cannabis abuse, 47.5% were found with schizophrenia, 20% with schizoaffective symptoms, 10% with manic symptoms, and 22.5% with both manic and psychotic symptoms. In the group–group and within-group statistical analysis, the results of thyroid hormones and cardiovascular parameters were non-significant. The psychiatric assessment has shown highly significant (*p* < 0.001) difference of positive, negative, general psychopathology, and total scores [through Positive and Negative Syndrome Scale (PANSS) rating scales] in patients vs. the healthy control subjects. The study revealed that cannabis abuse did not significantly alter thyroid hormones and cardiovascular parameters due to the development of tolerance. However, the cannabis abuse might have a significant contributing role in the positive, negative, and manic symptoms in different psychiatric disorders.

## Introduction

Substance-induced psychosis (SIPs) is a condition in which psychosis begins in the context of substance used and persists for days to weeks in the absence of that agent(s). The substances with psychoactive properties include cannabis, amphetamine, cocaine, lysergic acid diethylamide (LSD), phencyclidine, ketamine, benzodiazepines, and alcohol (1).

Cannabis is a widely used addictive agent after tobacco and alcohol. It has a prevalence of 3.6% of the local population in Khyber Pakhtunkhwa, Pakistan (2). Cannabis psychosis arises after consumption of a large quantity of cannabis. It is characterized by a number of symptoms, such as hallucinations, misidentifications, delusions and/or ideas of reference (often of a paranoid or persecutory nature), and psychomotor disturbances. These indications begin with the use of cannabis intoxication but continue beyond its usage (3, 4).

Tetrahydrocannabinol (THC) is a major constituent present in cannabis, which can initiate psychotic-like symptoms in its users. The severity and frequency of these symptoms are closely related to its usage. THC can produce brief psychotic symptoms through dopaminergic dysfunction and memory impairment through stimulation of cannabinoid receptor-1 (CB1R) (5). Experimental studies have uncovered that cannabis, THC, and synthetic cannabinoids can produce brief positive, negative, and cognitive symptoms in healthy volunteers (6). These synthetic cannabinoids are more potent than natural cannabis, since they act as a full agonist at their cannabinoid subtype-1 receptor as compared to the tetra-hydrocannabinol. They also lack cannabidiol that may otherwise counteract the psychoactive potency of THC. These synthetic cannabis compounds may induce a more severe symptomatic presentation, including anxiety, hallucination, tachycardia, memory and cognitive impairment, violent behavior, and psychosis. They are also reported to be associated with a variety of positive and negative symptoms and cognitive impairment that resemble the phenomenology of schizophrenia (7).

THC, the main psychoactive constituent present in cannabis, acutely alters several hormones including suppression of luteinizing hormone (8), testosterone (9), and thyroid hormone (TH), [T3] (10) and elevation of cortisol level (8). Cannabinoids also suppress the hypothalamic–pituitary–thyroid axis (HPA) at the pituitary level and thyroid gland (11). In the HPA axis, the basic hypothalamic hormone (TRH) causes synthesis in the anterior pituitary, resulting in the release of thyrotropin or thyroid stimulating hormone (TSH). TSH after entering into circulation activates the thyroid gland that causes a release of T3 and T4 hormones. Unbound T3 and T4 inhibit TRH release in the hypothalamus and TSH in the anterior pituitary through feedback mechanism. Hypothalamic Pituitary Thyroid (HPT) is then regulated by the negative feedback mechanism (11–13).

Acute exposure to cannabis smoking in humans can cause unfavorable consequences on the functions of the cardiovascular (CV) system. It causes an increase in the heart rate (HR), an increase in cardiac output (CO), reduced peripheral vascular resistance, and an overall moderate increase in blood pressure (BP), and sometimes postural hypotension. These deviations are mediated centrally by the autonomic nervous system (ANS) and peripherally by cannabinoid receptors mediated by vasodilatation. For many psychoactive drugs, including cannabis, the development of dependence is usually related to the tolerance, which develops when people are exposed to high doses for a sustained period. Tolerance to the acute CV effects of cannabis smoking develops over several days to a few weeks, but it is quickly lost when cannabinoid administration is stopped (14). A recent human positron emission tomography (PET) study also found that tolerance occurs due to regional brain downregulation/desensitization of CB1R in chronic cannabis smokers (15). Furthermore, the effects of cannabis depend on the mode of administration, time of use, the dose received, and the premorbid personality of the user (16).

In view of the above-mentioned facts and considering the higher percentage of drug abuse, addiction, dependence, and development of related psychotic disorders, this study was designed not only to evaluate the variety, severity, and multiplicity of psychotic symptoms possibly contributed due to cannabis abuse but also to find out the significant role of cannabis dependence to alter the selected THs and CV parameters in human subjects/patients.

## Materials and Methods

### Ethical Approval

This multicenter study was conducted at various tertiary care hospitals including Sarhad Hospital for Psychiatric Diseases, Lady Reading Hospital, Peshawar, Pakistan, and Syed's Clinic for Psychiatric Diseases, Peshawar, Pakistan. This study was conducted under the guidance of professionally renowned psychiatrists of Peshawar region. The protocol for the said study was formally approved by the Institutional Review Board of the Lady Reading Hospital, Peshawar, Pakistan, vide reference No: 10, dated June 1, 2015, and Department of Pharmacy, University of Peshawar *via* testament no: 08/EC-16/Pharm, dated July 12, 2015. This study was conducted in compliance with the principles of the declaration of Helsinki and its amendments (17).

### Study Design

Well-informed consent was obtained from the selected individuals (or their guardians, where applicable) at the beginning of the study. Drug use was determined by urine screening test, and all the individuals went through clinical examination for diagnosis regarding psychiatric symptoms. These symptoms were positively diagnosed on the basis of Diagnostic and Statistical Manual of Mental Disorders (DSM) IV (adopted) criteria by professional psychiatrists. The included subjects were then categorized into patients/test group (*n* = 40) and healthy control subjects (*n* = 40). Both groups were assessed on the basis of psychiatric, biochemical, and CV parameters.

### Inclusion and Exclusion Criteria

According to the inclusion criteria, new cannabis-related psychosis or relapse cases (*n* = 40) were selected after physical examination, clinical investigation, and previous medication history. Male patients of the age group ranging from 18 to 60 years, who used only cannabis during the preceding 30 days, were selected for the said study. Subjects with no family history of psychosis or any other psychotic disorders were also included in the study. However, those patients who were not willing to sign the informed consent form regarding participation in the study and patients having any history of serious illness like endocrinological, renal, neurological, hepatic, or thyroid abnormality were excluded from the study. Similarly, patients with a history of psychotic symptoms induced by drugs other than cannabis were also excluded from the study. In contrast, healthy control subjects were included if they had negative reports for cannabis abuse through urine screening/test; did not show any psychotic symptoms during assessment through Hamilton Depression Rating Scale (HAM-D-17), Positive and Negative Syndrome Scale (PANSS), and Young Mania Rating Scale (YMRS); and had normal thyroid hormonal levels and CV parameters. They were also not using any other kind of drug of abuse.

### Urine Screening

Patients were screened to determine the active use of drugs (i.e., cannabis) through multiple drug screening strips (Abon®, China) (18). This method was based on the immunoassay technique, used for the qualitative detection of cannabis or combination with other drugs in the specimen of human urine. After placing the multi-drug strip on a clean and leveled surface, three to four drops of urine were transferred to each portion(s) of the test strip from the dropper. The appearance of a colored line on the strip indicated a positive result. Based on test results, only the patients (*n* = 40) with positive screening for cannabis were selected for the study. Patients with positive results for substances other than cannabis were excluded from the sample. The healthy control subjects (*n* = 40) were also screened to ensure that the selected sample was of non-cannabis users.

### Serum TSH, T3, and T4 Quantification

About 4–5 ml of blood was collected from subjects of the study and then transferred to gel clot-activator tubes. These were centrifuged at 4,500 rpm for 10 min, and the serum part was separated for analysis. Biochemical variables TSH, T3, and T4 were analyzed using the commercial Roche diagnostics test kits (Roche®) by ELECYS 2010, following the chemiluminescence method. The reference ranges for TSH, T3, and T4 were adopted according to the guidelines of the National Academy of Clinical Biochemistry NACB which were 0.27–4.2 U/ml, 80–200 ng/dl, and 5.1–14.1 μg/dl, respectively (19).

### Physiological Assessment for Cardiovascular Parameters

Systolic and diastolic BP was measured by using a sphygmomanometer (Yamasu, Kenz medico®, Japan), and HR was calculated as beats per minute (bpm) using a stethoscope and stopwatch. According to the guidelines of the American Heart Association (AHA), BP values greater than 140/90 mmHg and <90/60 mmHg were considered hypertension and hypotension, respectively (20, 21). For HR, values above 100 bpm and below 60 bpm were considered indicating tachycardia and bradycardia, respectively (22). Subjects suffering from hypertension and hypotension or tachycardia and bradycardia symptomatic subjects were excluded from the study.

### Psychiatric Assessment

Included subjects were assessed for the severity of depressive, schizophrenic, and manic symptoms using HAM-D-17, PANSS, and YMRS, respectively, by professional psychiatrists.

### Assessment of Tolerance Development

The development of tolerance for cannabis was assessed by considering various parameters including dose, frequency, duration, route, and cannabis alone or in combination with other substances. The amount of cannabis consumed was quantified as grams per day. The frequency was calculated based on the intake a number of times daily, duration in years/months, and the route by using either joint or bong. It was also considered whether they have used any other drug of abuse in the past along with cannabis or not.

### Data Analysis

Participant's data (i.e., patients vs. healthy control subjects) were analyzed for the effect of cannabis on alteration of THs (TSH, T3, and T4), CV physiological parameters (systolic BP, diastolic BP, and HR), and to determine the severity of psychotic symptoms by using PANSS, YMRS, and HAM-D-17 scale. The parametric data from participants were further processed statistically using computer-based Prism Graph Pad (version 8). All results were expressed as mean ± SD. The non-paired analysis of variance (ANOVA) test was applied for the determination of the significance of the difference between means of respective parameters (i.e., *p* ≤ 0.05, two tails). The relation between marijuana consumption and the development of tolerance was assessed on the basis of factors including dose, frequency, duration, route, or cannabis alone or in combination. Finally, the results were correlated with the development of psychotic symptoms, fluctuation of THs, and CV physiological parameters in included patients.

## Results

Considering the significance and theme of the study, a total of 40 patients and 40 healthy control subjects were assessed to determine the effect of cannabis abuse and dependence on THs, CV physiological parameters, and its association with various psychotic symptoms by using PANSS, YMRS, and HAM-D-17.

The general demographic information about all the included participants is shown in Table 1 for both the healthy control subjects and the test group. Among the 40 patients using cannabis, 19 (47.5%) were diagnosed with cannabis-related schizophrenia, 8 (20%) with schizoaffective symptoms, 4 (10%) with manic symptoms, and 9 (22.5%) with both manic and psychotic symptoms.

**Table 1:** Demographic details of healthy control subjects vs. test group who participated in the study regarding psychiatric, biochemical, and physiological evaluation.

**Demographic parameters**	**Healthy control subjects** **(***n*** = 40)**	**Test group** **(***n*** = 40)**
Gender	Male	Male
**Age (years)**		
18–20	7 (17.5%)	9 (22.5%)
21–25	15 (37.5%)	13 (32.5%)
26–30	8 (20%)	8 (20%)
31–35	7 (17.5%)	5 (12.5%)
36–40	3 (7.5%)	5 (12.5%)
**Marital status**		
Single	25 (62.5%)	23 (57.5%)
Married	15 (37.5%)	17 (42.5%)
**Education**		
Illiterate	17 (42.5%)	26 (65%)
Matriculate	18 (45%)	13 (32.5%)
Graduate	5 (12.5%)	1 (2.5%)
**Forensic history**		
Positive	13 (32.5%)	7 (17.5%)
Negative	27 (67.5%)	33 (82.5%)
**Employment status**		
Manual work/labor	25 (62.5%)	7 (17.5%)
Student	5 (12.5%)	4 (10%)
Not working	10 (25%)	29 (72.5%)
**Socioeconomic status**		
Low	18 (45%)	23 (57.5%)
Satisfactory	13 (32.5%)	11 (27.5%)
Good	09 (22.5%)	8 (20%)
**Smoking status**		
Light smokers (1–4 cigarettes per day)	14 (35%)	24 (60%)
Medium smokers (14–24 cigarettes per day)	18 (45%)	12 (30%)
Heavy (25 or more per day)	8 (20%)	4 (25%)

The harmful effect of cannabis use and dependence on THs TSH, T3, and T4 are presented in Table 2. The results were non-significant (i.e., *p* > 0.05), representing that there was no impact on THs. Results regarding the biochemical evaluation for TH (Table 3) were also statistically non-significant when patients were compared within group (schizophrenia, schizoaffective symptoms, mania with psychosis, and mania).

**Table 2:** Effect of cannabis abuse and/or dependence on thyroid hormones (TSH, T3, and T4).

	**Healthy control subjects** **Mean ± SD** **(***n*** = 40)**	**Test group** **Mean ± SD** **(***n*** = 40)**	**Healthy control subjects** **Max (Min)** **(***n*** = 40)**	**Test group** **Max (Min)** **(***n*** = 40)**	***P*** **-value**
TSH (IU/ml)	1.30 ± 0.85	1.27 ± 1.09	2.70 (0.18)	5.43 (0.005)	0.46
T3 (ng/dl)	111.3 ± 20.54	108.3 ± 29.27	136 (67.95)	189 (69.58)	0.38
T4 (μg/dl)	7.47 ± 1.32	8.55 ± 1.96	9.47 (4.60)	12.74 (5.19)	0.06

*Data presented as mean ± SD*.

**Table 3:** Biochemical evaluation of healthy control subjects vs. test group, i.e., patients with cannabis use and psychotic symptoms (schizophrenia, schizoaffective symptoms, mania with psychosis, and mania).

**Parameters**	**Healthy control subjects** **(***n*** = 40)**	**Schizophrenia** **(***n*** = 19)**	**Schizoaffective symptoms** **(***n*** = 8)**	**Mania with psychosis** **(***n*** = 9)**	**Mania** **(***n*** = 4)**	***P*** **-value**
TSH value	1.30 ± 0.85	1.61 ± 1.38	1.047 ± 0.92	0.82 ± 0.42	1.09 ± 0.39	0.39
T3 value	111.3 ± 20.54	115.0 ± 34.21	111.1 ± 30.02	95.33 ± 19.04	100.0 ± 12.21	0.47
T4 value	7.47 ± 1.32	8.82 ± 2.49	8.51 ± 1.36	8.034 ± 1.56	8.55 ± 0.94	0.50

*Data presented as mean ± SD*.

Similarly, consequent upon performing the group-to-group and within-group statistical analysis, the effect of cannabis use on CV physiological parameters (i.e., systolic BP, diastolic BP, and HR) were also non-significant (*p* > 0.05), showing no impact on CV physiological parameters (Table 4).

**Table 4:** Evaluation of healthy control subjects vs. test group, i.e., patients with cannabis use and psychotic symptoms (schizophrenia, schizoaffective symptoms, mania with psychosis, and mania) for selected cardiovascular parameters (systolic BP, diastolic BP, and HR).

**Parameters**	**Healthy control subjects** **(***n*** = 40)**	**Schizophrenia** **(***n*** = 19)**	**Schizoaffective symptoms** **(***n*** = 8)**	**Mania with psychosis** **(***n*** = 9)**	**Mania** **(***n*** = 4)**	***P*** **-value**
Systolic BP	120.0 ± 7.07	116.3 ± 10.65	120.0 ± 9.25	118.9 ± 3.33	115.0 ± 12.91	0.73
Diastolic BP	80.00 ± 5.00	76.32 ± 7.60	75.00 ± 7.55	77.78 ± 4.41	75.00 ± 10.10	0.51
Heart rate	72.33 ± 2.00	76.95 ± 8.50	74.25 ± 7.26	73.78 ± 6.41	71.75 ± 2.36	0.40

*Data presented as mean ± SD*.

**Figure 1:**
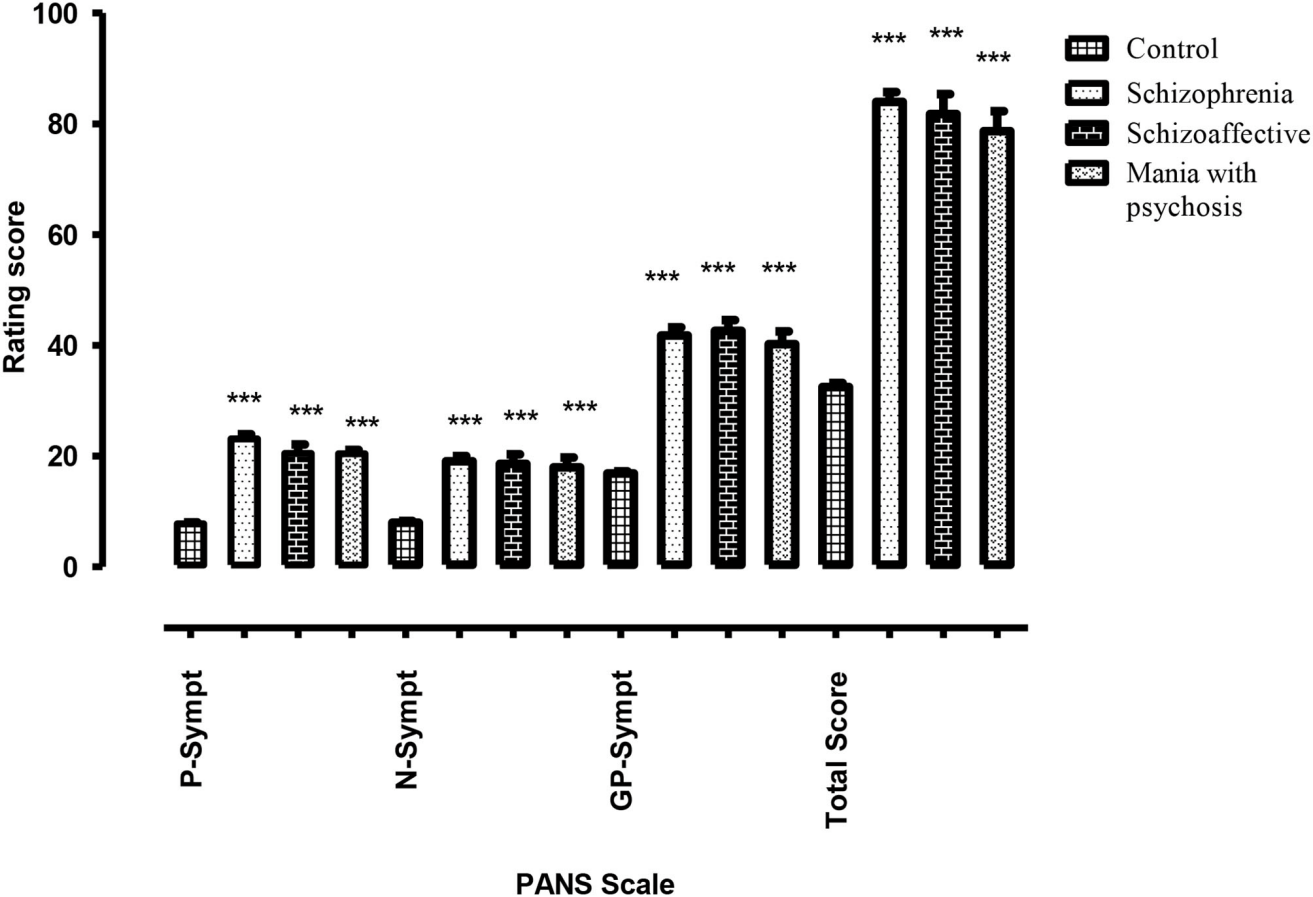
Difference of psychotic symptoms assessed through PANSS (positive, negative, general psychopathology, and total sub-scale) in healthy control subjects vs. test group of psychotic patients with cannabis abuse (schizophrenia, schizoaffective symptoms, and mania with psychosis). *** highly significant.

In case of psychiatric manifestations, the positive, negative, general psychopathology (GP), and total scores of PANSS for the patient group were found to be statistically highly significant (*p* < 0.001) when compared with the score of healthy control subjects. However, within the same patient group, the results were statistically non-significant (*p* > 0.05) as shown in Figure 1 and Table 5.

**Table 5:** Psychiatric evaluation of healthy control subjects vs. test group, i.e., patients with cannabis use and psychotic symptoms (schizophrenia, schizoaffective symptoms, mania with psychosis, and mania).

**Parameters**	**Healthy control subjects** **(***n*** = 40)**	**Schizophrenia** **(***n*** = 19)**	**Schizoaffective symptoms** **(***n*** = 8)**	**Mania with psychosis** **(***n*** = 9)**	**Mania** **(***n*** = 4)**	***P*** **-value**
PANSS positive score	7.77 ± 0.83	23.11 ± 3.72	20.50 ± 4.59	20.44 ± 2.00	-	<0.0001
PANSS negative score	8.00 ± 0.86	19.05 ± 4.19	18.63 ± 4.86	18.00 ± 5.29	-	<0.0001
PANSS general psychopathology score	16.89 ± 1.05	41.79 ± 6.34	42.63 ± 5.52	40.22 ± 6.83	-	<0.0001
PANSS total score	32.44 ± 2.29	83.94 ± 7.79	81.75 ± 10.22	78.67 ± 10.84	-	<0.0001
HAM-D-17	2.00 ± 0.0	-	13.75 ± 2.60	-	-	-
YMRS	4.00 ± 0.0	-	-	29.22 ± 2.72	31.75 ± 3.20	<0.0001

*Data presented as mean ± SD*.

^***^p < 0.001.

Based on psychiatric assessment among patients diagnosed with schizophrenia, about 57.89% of the patients exhibited higher score in positive symptoms. Moreover, results for ~31.75% of the patients reflected higher intensity of negative symptoms. However, few cases, i.e., 10.52%, revealed greater GP symptoms.

In the schizoaffective group, 62.5% of the patients reflected higher score in positive symptoms, while 25% of the patients have shown greater intensity of negative symptoms. Very few cases, i.e., 12.5%, revealed severe symptoms of GP. Further, the results indicated that among these patients, the majority, i.e., 62.5%, represented the symptoms of mild depression, whereas the remaining, i.e., 37.5%, were with moderate depression.

Similarly, out of those patients diagnosed with mania along with psychotic symptoms, 55.55% of the subjects demonstrated higher positive symptoms, whereas 33.33% have shown greater negative symptoms. There was only one case with higher score in GP symptoms. The results also indicated that 10% of the patients have shown symptoms of mania but not psychosis.

As chronic cannabis use can lead to tolerance development, it was assessed by considering the factors including duration, dose, frequency, route, and cannabis alone or in combination with other substances (Table 6). The results indicated that the duration, dose, and frequency of cannabis use were 8.13 ± 4.17 years, 2.73 ± 0.80 g/day, and 2.25 ± 0.74 times per day, respectively.

**Table 6:** Cannabis-related variables for tolerance assessment.

**Variables**	**Percentages (%)**
**Dose of cannabis used per day (g)**	
<1 g	3 (7.5)
1–2 g	9 (22.5)
2–3 g	27 (67.5)
3–4 g	6 (15)
**Duration of cannabis used (years)**	
0.5–5	13 (32.5)
6–10	14 (35)
11–15	9 (22.5)
Above 15	1 (2.5)
**Route (smoke)**	
**Method of use**	
Joint	19 (47.5)
Bong	8 (20)
Both	13 (32.5)
**Frequency used per day**	
Once	6 (15)
Twice	18 (45)
Three times and above	15 (37.5)
**Usage of drugs of abuse in the past**	
Cannabis alone	31 (77.5)
**Previous recreational exposure to illicit drugs other than cannabis**	
Alcohol	4 (10)
Heroine	2 (5)
Opium	1 (2.5)
Opium/heroine/alcohol	2 (5)
Tobacco use	Regular

## Discussion

The results have shown that TSH, T3, and T4 levels observed in all the patients were within the normal range of reference. This shows that continuous exposure to cannabis has no significant impact on TH levels and CV parameters due to development of tolerance. This is evident from earlier studies also, where exposure to cannabis twice a day for 14 consecutive days reduced the thyroid alteration effect in rats. The downregulation of CB1R in the central nervous system (CNS) is the underlying cause for the development of tolerance to cannabinoids. These are organized along the central and peripheral TH axis and with greater magnitude in the hippocampus and cerebellum regions of the brain (23). The results from our study were in concordance with TSH, T3, and T4 as presented by Bonnet (24). However, the study reported by Herning et al. (25) showed the lower value of T4.

Biochemically, the present results also revealed that 15% of the patients had lower T3 but normal TSH and T4 values, suggesting euthyroid sick syndrome. Furthermore, we have observed 10% sub-clinical hyperthyroidism and 5% sub-clinical hypothyroidism. Such dispersed results might be an indicator that the study sample had certain risk factors of thyroid dysfunction resulting from treatment provided to those included patients. Similar effects have also been reported in earlier studies, suggesting that in patients with psychiatric symptoms especially bipolar mania, treatment with lithium and haloperidol may have an alteration effect on serum TSH level (26).

It is important to note that smoking tobacco may either have a weak stimulatory or no effect on thyroid function. According to earlier studies, nicotine-induced sympathetic activation could account for small increases in THs main serum T3 and TSH levels (27).

Furthermore, statistically non-significant effects of cannabis dependence on CV physiological parameters (systolic BP, diastolic BP, and HR) have been indicated by the results of the investigation. This might be due to the fact that the use of cannabis leads to dose-dependent increased HR, increased BP, and CO, but sometimes it causes reduced peripheral vascular resistance and postural hypotension. The CV effects are modulated centrally by ANS and peripherally by the cannabinoid receptor system (28).

Moreover, the CV response to cannabis dose is biphasic in nature. A low to moderate dose of cannabis causes an increase in sympathetic activity with a reduction in parasympathetic activity. This results in marked tachycardia and an increase in CO (as much as 30%) and an overall increase in BP. On the other hand, high doses of cannabis lead to a decrease in sympathetic activity with increased parasympathetic activity. This results in bradycardia and hypotension (29–31). The exact mechanism of change in HR is unclear yet. However, it has been suggested that β-adrenergic blockade attenuates HR increase, and co-administration of a cannabinoid receptor antagonist also blocks the tachycardia, proposing that CB1R plays a vital role in alteration in HR and BP (32).

Decrease in both systolic and diastolic BP due to chronic use of cannabis has been reported in various studies (15, 28), whereas a decrease in diastolic BP but not in systolic BP has been observed by Schwope et al. (33). Similarly, an increase in HR had also been reported in a study conducted by Gorelick et al. (15). Development of tolerance to the acute effects of marijuana smoking might contribute to the stronger and more consistent effects in regular or chronic users of marijuana (28).

The psychiatric assessment revealed overall higher intensity for positive symptoms in psychotic/schizophrenic patients. The results were similar for schizoaffective patients as well. However, when compared with schizophrenics, the results indicated lower scores for PANSS positive and negative symptoms.

The patients suffering from mania and psychosis and who were cannabis abusers have shown high intensity of positive symptoms. However, the scores for positive, negative, and GP as well as of the total PANSS score were lower than that of schizophrenic and schizoaffective patients. Furthermore, some of the patients showed only the symptoms of mania and not psychosis.

D'Souza et al. (3) explained that THC affects dopamine neurotransmission in several regions of the brain, including the prefrontal cortex (PFC) and mesolimbic regions. THC-mediated increased mesolimbic dopaminergic activity *via* CB1R activation provides a possible reason for the increase in positive psychotic symptoms induced by cannabis.

The CB1R activation in the PFC might be responsible for the cognitive deficits and negative symptoms of cannabis. Moreover, cannabinoids modulate the release of neurotransmitters such as dopamine, γ-aminobutyric acid (GABA), and glutamate. By suppressing GABAergic and dopaminergic inhibitory neurotransmission, cannabinoids contribute to worsen the working memory effects and negative symptoms as observed in schizophrenia (34).

Moreover, it has also been reported that THC increases glutamate level in PFC which in turn leads to psychoactive and behavioral effects of marijuana consumption (35). Since the results also indicated that harmful use of cannabis in schizophrenia is related to the lower intensity of negative symptoms, especially when cannabis is the only drug abused and the user just starts before the onset of schizophrenia, it can be presumed that cannabis use might be a major factor for schizophrenia (36).

Cannabis-related increased intensity for positive symptoms but low for negative symptoms in the case of schizophrenia has also been supported by studies (18, 37). However, the results have not been supported by previous studies (38, 39) which indicated an increase in the intensity of negative symptoms but less of positive symptoms.

Assessment of the patients by HAM-D-17 indicated mild depression in cannabis abuse subjects. Similarly, pre-clinical and clinical studies have suggested that cannabis causes an increase in 5-HT (serotonin) neurotransmission in the PFC due to the activation of CB1R which modifies the mood of those subjects (40, 41). Furthermore, it is also suggested that cannabinoids inhibit the re-uptake of serotonin, not epinephrine and dopamine, and possess integral pharmacological properties similar to antidepressant drugs (42). The same results were also observed in a study conducted by Tosato et al. (43). In another clinical study, sub-clinical depression related to the harmful use of cannabis was observed along with the presence of psychotic symptoms (44). The results were not supported by the study conducted by Degenhardt et al. (45), where he found statistically significant increase in symptoms of psychosis.

As cannabis abuse may aggravate both psychotic and manic symptoms, it is suggested to share genetic susceptibility to dysregulation of the dopaminergic system due to stress induced by cannabis (5). Brain imaging and pharmacological studies suggest that dopaminergic hyperactivity induced by cannabis is the underlying cause for both psychosis and mania (46, 47). In addition, CB1R also reduces the uptake of dopamine, therefore further potentiating its effects (3). These suggest that chronic and heavy use of cannabis might contribute to the development of mania-like psychosis by sensitization of the dopaminergic system (48). Furthermore, increase in glutamate level mediated by THC in PFC also resulted in the development of manic symptoms in patients with acute bipolar disorder (BD) (49).

The findings of the study were supported by previous studies conducted by Kumar et al. (50) and Stone et al. (51), which stipulated similar findings, i.e., increased positive and manic symptoms due to cannabis use. Further increase in manic symptoms but controlled psychotic symptoms at baseline was observed in a study conducted by Henquet et al. (48).

Previous studies also revealed that THC might have significant psychotomimetic effects and perceptual alterations. These effects were dose-dependent and subjectively high as assessed through PANSS rating scale (6). It is likely that increase in heart and pulse rate is an important factor to judge the degree of cannabis intoxication. However, unlike physiological effects, the development of tolerance is not reflective in the subject's psychosis. Therefore, cannabis use seems to exert its effects through increased dopaminergic activity, which is a common pathway responsible for the worsening of positive psychotic and manic symptoms (52).

## Study Limitations

Despite strict inclusion and exclusion criteria and dedicated efforts to conduct this multicenter research, the study would be statistically more meaningful if executed on a relatively large population and included with both male and female genders. Similarly, it would have a great impact if the effects due to acute vs. chronic cannabis abuse were compared. Furthermore, follow-up evaluation of the subjects would have indicated more evident information regarding the exacerbation in the psychotic symptoms.

## Conclusion

Cannabis dependence had a non-significant impact on serum THs (TSH, T3, and T4) and CV physiological parameters (systolic BP, diastolic BP, and HR). This could be due to the development of tolerance resulting from the downregulation of CB1R. This study further revealed that cannabis might have association with the positive, negative, and manic symptoms in different schizophreniform, schizoaffective symptoms, manic disorders, and mild depression.

## Data Availability Statement

The original contributions presented in the study are included in the article/supplementary material, further inquiries can be directed to the corresponding author/s.

## Ethics Statement

The studies involving human participants were reviewed and approved by Institutional Review Board of the Lady Reading Hospital, Peshawar, Pakistan vide reference No: 10. Dated; 01.06.2015 and Department of Pharmacy, University of Peshawar *via* testament no: 08/EC-16/Pharm. Dated 12.07.2015. The patients/participants provided their written informed consent to participate in this study.

## Author Contributions

AM performed all the experiments and wrote the manuscript. SU, FS, and ZN supervised the research team and edited the manuscript. SH, FK, and AKha participated in the data interpretation. AKhu, MUKS, and SA performed literature study and data compilation. AE-S and TE provided the technical assistance. BJ and JA reviewed and edited the manuscript. All authors have read and approved the manuscript.

## Funding

The current work was funded by Taif University Researchers Supporting Project Number (TURSP-2020/202), Taif University, Taif, Saudi Arabia.

## Conflict of Interest

The authors declare that the research was conducted in the absence of any commercial or financial relationships that could be construed as a potential conflict of interest.

## Publisher's Note

All claims expressed in this article are solely those of the authors and do not necessarily represent those of their affiliated organizations, or those of the publisher, the editors and the reviewers. Any product that may be evaluated in this article, or claim that may be made by its manufacturer, is not guaranteed or endorsed by the publisher.
